# AHAK: a new effective medical countermeasure against percutaneous intoxication by Novichok chemical warfare agents

**DOI:** 10.1007/s00204-026-04355-6

**Published:** 2026-04-20

**Authors:** Uri Nili, Victoria Nahum, Boris Smolkin, Nissan Ashkenazi, Eugenia Bloch-Shilderman

**Affiliations:** 1 Department of Pharmacology, IIBR – Israel Institute for Biological Research, P.O. Box 19, 7410001 Ness Ziona, Israel; 2 Department of Organic Chemistry, IIBR – Israel Institute for Biological Research, P.O. Box 19, 7410001 Ness Ziona, Israel

**Keywords:** Novichok A-232, Acetohydroxamic acid (AHA), AHAK, “Catch-up” therapy, Dermal depot, Low-volatility OP CWAs, Percutaneous intoxication/poisoning/exposure, VX, Skin decontamination

## Abstract

Novichoks are the latest known and most toxic class of organophosphorus chemical warfare agents (OP CWAs) to have been developed. Among the documented Novichoks, A-232 is the most toxic and most potent inhibitor of AChE, as well as the most resistant to reactivation by oximes, and thus likely the most challenging to treat. Here we characterize the intoxication following dermal exposure to the Novichok A-232 in swine, and describe a novel countermeasure against it. Our results demonstrate that percutaneous poisoning by a lethal dose of A-232 (2 mg/kg) is characterized by slow appearance and exacerbation of intoxication signs. We further show that conventional antidotal treatment against such poisoning, administered upon appearance of intoxication signs, provides only temporary relief after which intoxication signs reoccur, and that further repeated such treatment is not sufficient to enable survival. The above likely stem from formation of a dermal depot of A-232 from which the agent slowly penetrates into the bloodstream, coupled with its resistance to reactivation by oximes. Accordingly, we developed the AHAK lotion (potassium acetohydroxamate in DMSO/H_2_O, derived from the FDA approved medication acetohydroxamic acid (AHA)), intended to act as a ‘catch-up therapy’ enabling both skin surface and intradermal decomposition of persistent low-volatility OP CWAs. We demonstrate that combining conventional antidotal treatment with dermal application of AHAK following percutaneous poisoning by a lethal dose of A-232, leads to survival of all animals. Additionally, using a low dose (0.33–0.41 mg/kg) of A-232, we show that AHAK is expected to exert a beneficial effect also following late application. Hence, the results of the current study, coupled with our previous results regarding the contribution of AHAK to countering percutaneous intoxication by VX and the safety of prolonged whole-body application of AHAK, delineate the aptness of AHAK to serve as a generic medical countermeasure against percutaneous intoxication by persistent low-volatility OP CWAs, of both the V and Novichok families.

## Introduction

Organophosphorus (OP) chemical warfare agents (CWAs) are extremely toxic substances that irreversibly inhibit the enzyme acetylcholinesterase (AChE). This inhibition causes accumulation of the neurotransmitter acetylcholine in synaptic clefts, leading to overstimulation of cholinergic receptors and a range of toxic effects. These include increased tracheobronchial secretions, bronchial constriction, convulsions, respiratory depression, and ultimately death (Munro et al. 1994; Hartmann 2002; Marrs 2007).

Novichoks are a class of highly toxic low-volatility persistent OP CWAs, developed by the Soviet Union during the Cold War in the 1970s and 1980s (Mirzayanov 2008). The name “Novichok” translates to “newcomer” in Russian, reflecting the status of the Novichoks as newer OP CWAs compared to the more familiar older generation OP CWAs of the G- (e.g., sarin and soman. ) and V-series (e.g., VX and RVX ). It has been suggested that Novichoks are significantly more toxic and challenging to treat than the older generation OP CWAs (Bolt et al. 2022; Blom et al. 2023), and accordingly pose a serious threat in the context of chemical warfare, acts of terror or targeted assassinations. Indeed, the Novichoks gained widespread international attention following their recent use in two high-profile poisoning incidents (Salisbury and Amesbury, UK, 2018 (OPCW); Poisoning of Alexei Navalny in 2021, (Steindl et al. 2021)), the first of which also delineated the extremely long environmental persistence and difficulty of decontamination of these CWAs (Vale et al. 2018; Columbus et al. 2025).

In a real-life exposure scenario, volatile CWAs such as sarin or soman will most likely exert their toxicity mainly by inhalation, which will lead to rapid onset of cholinergic symptoms due to quick absorption through the respiratory system (Munro et al. 1994). In contrast, low-volatility OP CWAs are expected to exert their toxicity primarily by skin penetration, which as has been demonstrated for VX will highly likely result in a much slower onset of symptoms (van der Schans et al. 2008; Joosen et al. 2010, 2017; Thiermann et al. 2016; Bloch-Shilderman et al. 2019, 2023). Furthermore, it has been demonstrated that if skin decontamination following dermal VX exposure is delayed, antidotal treatment might provide only temporary relief, and recurrence of toxic signs may necessitate repeated administration of antidotes (Joosen et al. 2010, 2013; Bloch-Shilderman et al. 2019, 2023). This phenomenon has been postulated to stem from a faster absorption of VX into the skin than from the skin to the bloodstream, leading to formation of a dermal depot of the agent from which it slowly and continuously penetrates into the circulation and on to target organs such as the diaphragm and the brain (Chilcott et al. 2005a, b; Wetherell et al. 2008; Schwartz et al. 2012; Joosen et al. 2010, 2013, 2017; Bloch-Shilderman et al. 2023). In the case that dermal exposure to Novichoks will also lead to formation of a dermal depot, as has been shown for VX, their high stability against hydrolysis at physiological pH (Harvey et al. 2020; deKoning et al. 2022; Smolkin et al. 2024) might result in sustained high levels of these CWAs in the bloodstream. This, coupled with their high resistance to oxime treatment (Steindl et al. 2021; Hrabinova et al. 2024; Kovarik et al. 2025), will highly likely lead to prolonged systemic ChE inhibition. This in turn may necessitate prolonged intensive medical intervention such as long periods of mechanical ventilation, or uncommon practices in the field of CWA casualty management such as plasma replacement (Steindl et al. 2021), to enable survival.

Traditionally, medical protocols to counter the toxicity of OP CWAs consist of physical removal of the agent from the skin surface as soon as possible (Braue et al. 2011; Schwartz et al. 2012; Thors et al. 2017; Hulse et al. 2019), and administration of antidotes in the case of intoxication signs (Hamilton et al. 2004; Thiermann et al. 2016). However, as described above, in a real-life scenario, intoxication signs following percutaneous exposure to persistent low-volatility OP CWAs, which will most likely be the trigger to treat, may be long delayed (Nozaki et al. 1995; Steindl et al. 2021). Accordingly, by the time treatment will commence, a substantial amount of the agent will almost certainly already be absorbed into the skin forming a dermal depot (Wetherell et al. 2008; Joosen et al. 2013), rendering decontamination of the skin surface only partially effective (Chilcott et al. 2005a; Wetherell et al. 2008; Schwartz et al. 2012; Joosen et al. 2013; Bloch-Shilderman et al. 2023) and necessitating repeated administration of antidotes (Joosen et al. 2010, 2013; Bloch-Shilderman et al. 2019; 2023).

In light of the above, we developed an active decontamination lotion consisting of the potassium salt of acetohydroxamic acid (AHA) in DMSO/H_2_O 1:4 (hereafter AHAK), intended for both skin surface and intradermal persistent low-volatility OP CWA decomposition (Nahum et al. 2021). In our recent work we described the beneficial effect of the AHAK lotion when combined with existing medical countermeasures against the detrimental effects of VX dermal exposure in vivo, and its compatibility with prolonged whole-body dermal application (Bloch-Shilderman et al. 2023). Additionally, we demonstrated its high in-vitro efficacy in decomposing not only VX but also three of the most notable Novichoks (A-230, A-232 and A-234), with reasonably fast kinetic rates (Smolkin et al. 2024).

In the current study, we first characterized the intoxication following dermal exposure to a lethal dose of the Novichok CWA A-232, known for its high toxicity and efficiency in inhibiting ChE (Harvey et al. 2020; Kovarik et al. 2025) and believed to have been adapted for military use (Mirzayanov 2008), in an awake (unanesthetized) swine model. We then examined the suitability of the AHAK lotion as a countermeasure against such exposure. The swine is one of the commonly accepted large animal models for the study of OP CWAs intoxication (Dorandeu et al. 2007). The swine is also considered as the most adequate animal model for human percutaneous absorption research (Simon and Maibach 2000; Chilcott et al. 2003; Dorandeu et al. 2007; Fémy et al. 2023), as porcine skin is similar to human skin in aspects such as skin structure and function (Debeer et al. 2013), as well as permeability characteristics (Bartek et al. 1972). Hence, the swine is especially suited for characterization of percutaneous poisoning by OP CWAs and medical countermeasures against it.

## Materials and methods

**Caution!** OP CWAs are extremely toxic compounds, with special emphasis on A-232. Experiments with these compounds should only be performed by trained personnel using appropriate applicable safety procedures.

### Chemicals

Methyl N-[1-(diethylamino) ethylidene] phosphoramidofluoridate (A-232) was synthesized in-house (> 95% purity). Acetohydroxamic acid (AHA) was purchased from Biosynth Carbosynth, UK. Acetylcholine iodide [Acetyl-^3^H] (specific activity 58.0 mCi/mmol) was purchased from Perkin-Elmer Life and Analytical Sciences Inc., MA, USA. Isoamyl alcohol was purchased from Merck, Darmstadt, Germany. QuickSafe scintillation liquid for the determination of ChE activity was purchased from Zinsser, Germany. Atropine sulfate, acetylcholine chloride, dimethyl sulfoxide (DMSO) and benactyzine were purchased from Sigma Chemical Co., St. Louis, MO, USA. TMB-4 was purchased from the Casali Center of Applied Chemistry, Hebrew University, Jerusalem, Israel. Midazolam was purchased from Rafa Laboratories, Ltd, Jerusalem, Israel. Sodium-chloride solution (saline, 0.9%) was purchased from TEVA Medical Ltd, Israel.

### AHAK

In the experiments described in this study, two AHAK solution variants with different concentrations of the potassium salt of AHA were utilized: 1. AHAK_220_ consisting of 220 mg/ml of the potassium salt of AHA in DMSO/H_2_O 1:4. 2. AHAK_880_ consisting of 880 mg/ml of the potassium salt of AHA in DMSO/H_2_O 1:4, the highest concentration of potassium acetohydroxamate that is soluble in the DMSO/H_2_O 1:4 solvents mixture. Additionally, in the AHAK_220_ efficacy evaluation experiment, a mock AHAK_220_ solution (hereafter AHAK_0_), consisting of DMSO/H_2_O 1:4 and a minute amount of curcumin extract in order to obtain a similar color to the AHAK_220_ mixture, was used. The AHAK_0_ solution was identical to the AHAK_220_ solution in terms of texture and color.

### Preparation of the AHAK_220_ and AHAK_880_ solutions

Preparation of AHAK_220_: Acetohydroxamic acid (7.88 gr, 104.72 mmol, 1 equiv.) and potassium hydroxide (5.45 gr, 97.25 mmol, 0.93 equiv.) were placed in a 100 ml glass bottle. DMSO (4.21 ml) and deionized water (15.1 ml) were added and the slurry was stirred until completely dissolved while cooled in an ice bath. The final volume of the solution was 50.0 ml, with a concentration of 220 mg/ml of potassium acetohydroxamate. Preparation of AHAK_880_ was identical to the above, with the respective amounts of acetohydroxamic acid (31.52 g, 418. 9 mmol) potassium hydroxide (21.82 gr, 389.0 mmol), DMSO (4.21 ml) and deionized water (9.84 ml), leading to a potassium acetohydroxamate concentration of 880 mg/ml. For more details see Bloch-Shilderman et al. (2023).

### Animals

Weaned female pigs (Topigs 20; 8.35–12.75 kg, n = 36) were obtained from van Beek, Netherlands, fed on standard pig diet (4% body weight food (Denkapigs) per day) and housed in a light and temperature-controlled environment (12 h light/dark cycle, lights on at 06:00, 22 ± 1 °C), in a designated animal facility, for 4–11 days prior to the start of the experiments. Environmental enrichment was provided. Animals were allowed ad libitum access to water. Food was withdrawn 12 h before the experimental procedure.

### Whole-blood ChE activity assays

Heparinized blood samples (100 μl), drawn from the jugular vein, were immediately diluted 13-fold in ice cold distilled water and frozen at – 70 °C until analysis. The samples were analyzed for ChE activity according to the modified radiometric method of Johnson and Russell (1975). Frozen samples were thawed and then diluted 1:1 in 0.2 M Tris/HCl buffer, pH 7.4. Triplet aliquots of 50 μl each were further diluted 1:1 with [3H] acetylcholine iodide and incubated at 25 °C for 30 min (the [^3^H] acetylcholine iodide solution was prepared by dilution with a freshly prepared solution of unlabeled acetylcholine chloride to provide the substrate at an appropriate concentration [6 nM]). Specific activity of the labeled substrate was 58 mCi/mmol. The reaction was terminated by adding 0.1 ml of stopping mixture (1 M chloroacetic acid, 0.5 M NaOH, and 2 M NaC1) followed immediately by 2.5 ml of scintillation mixture (QuickSafe commercial scintillation mixed with 10% isoamyl alcohol). Vials were capped and shaken. Radioactivity was measured using the 1600TR liquid scintillation analyzer (Packard, Meriden, CT, USA). The CPM value of a no-enzyme blank sample was subtracted from all CPM values. Results are expressed as % activity relative to baseline ([post-exposure CPM/basal CPM] × 100).

### Antidotes administration

All treated pigs were given TAB (the oxime TMB-4, 1.14 mg/kg; the cholinolytic atropine, 0.07 mg/kg; the CNS active cholinolytic benactyzine, 0.09 mg/kg) or TA (TMB-4, 1.14 mg/kg; atropine, 0.07 mg/kg) i.m. upon onset of obvious signs of intoxication (the exact times for the initial TAB/TA administration in all treated animals are given in the “Results” section). These two mixtures were tested as they are the ones utilized in the Israeli auto injectors intended for immediate emergency use against OP CWAs. The TAB mixture has been shown to provide significant protection against whole-body VX vapor exposure in the rat model (Bloch-Shilderman et al. 2019), as well as following percutaneous VX intoxication in the swine model (Bloch-Shilderman et al. 2023). All drug doses were calculated based on human equivalents as follows: Israeli TAB auto-injectors contain 80, 3 and 4 mg of TMB-4, atropine and benactyzine, respectively (the TA auto-injectors contain the same constituents, excluding benactyzine). These were divided by 70 (kg) to calculate a mg/kg equivalent. Doses of the cholinolytic agents were additionally factored by 1.6 for weight-to-body surface area ratio correction (Nair and Jacob 2016). In the AHAK_220_ efficacy evaluation experiment (see below), pending recurrence of intoxication signs after TAB administration, animals were given repeated i.m. administrations of TAB. This treatment regime was used, as it represents an optimal immediate treatment upon OP CWA intoxication signs appearance. Since following treatment animals seemed to recover (disappearance of symptoms) but then symptoms reoccurred, each such reoccurrence was regarded as “intoxication signs appearance”, following which the optimal treatment (TAB) was given.

In the AHAK_880_ efficacy evaluation experiment (see “AHAK_220_/AHAK_0_/AHAK_880_ efficacy evaluation experiments” below), pending recurrence of intoxication signs after TA or TAB administration, animals were treated with the standard continuous antidotal treatment approved in Israel, comprising of atropine and midazolam, excluding the use of the oxime obidoxime. This was due to previous demonstration of the inefficacy of this oxime in reactivation of AChE inhibited by Novichoks (un-published data; Steindl et al. 2021). Specifically, the animals were given repeated i.m. administrations of atropine (0.07 mg/kg, see dose rational above) until atropinization (dryness of the mouth and nose) and midazolam upon convulsions or loss of consciousness (0.22 mg/kg, a human mg/kg equivalent dose based on a human 10 mg dose, factored by 1.6 for weight-to-body surface area ratio correction (Nair and Jacob 2016)).

### AHAK_220_/AHAK_0_/AHAK_880_ dermal application

Immediately following administration of the initial TAB/TA treatment, the AHAK solution variant used (50 ml of either AHAK_220_**/**AHAK_0_**/**AHAK_880_) was slowly (in the course of ~ 10 min) applied to the skin surface at the site and the surroundings of A-232 exposure. This was done using a regular household sponge, to insure most of the lotion remained on the skin. Several hours after application, a solid salt deposit appeared on the treated skin, probably due to evaporation/penetration of the AHAK solvents. ~ 24 h post exposure the animal was washed to remove all remains of the lotion.

### Percutaneous A-232 exposure procedure

Unanesthetized pigs were restrained and their hair trimmed at the shoulders and the nape of the neck. The area of exposure (3X3cm) between the shoulder blades was marked. A baseline whole-blood sample (~ 200µl) was withdrawn from the jugular vein. Liquid (neat) A-232 was then applied by slow dripping (0.5 or 1.0 µl droplets) into the marked area (for instance, if an animal weighed 9.25 kg, as 1 µl of A-232 weighs ~ 1 mg, for a 2 mg/kg dose exposure (a total dose of 18.5 mg), 19 droplets (18 of 1.0 µl and 1 of 0.5 µl) were applied). Exposure initiation time was defined as the time at the end of A-232 application. A-232, similarly to VX, is odorless, colorless and a lethal percutaneous dose of the agent is expected to be very low (a few microliters - similar to VX, Munro et al. 1994). The above properties, coupled with expected slow dynamics of percutaneous intoxication by A-232 (as characteristic of VX, *ibid*; van der Schans et al. 2003), suggest that in a real-life scenario of dermal exposure to the agent, the probability of the agent residing on the skin long enough (hours) to cause intoxication without being identified is quite high, as has been reported in men for A-234 (Salisbury 2018). Accordingly, the maximal exposure duration was set to 3 h.

In the intoxication characterization and AHAK_220_ efficacy determination experiments (see “Study design” below), a procedure for physical removal of residual skin surface A-232 (hereafter skin surface physical decontamination (SSPD)) was performed at the end of the exposure. This included repeated wiping with dry (X5) followed by wet (ethanol soaked) gauze pads (X5) of the skin area applied with A-232, repeated twice, followed by an additional single wiping with a dry gauze pad (verification of the efficacy of this procedure in decontamination of residual skin surface A-232 by a GC analysis of skin swabs from the site of exposure, resulted in no residual A-232 detection following the procedure). Animals were then returned to designated cages for follow-up. In both the AHAK_220_ and AHAK_880_ efficacy determination experiments, antidotal treatment was initiated upon appearance of obvious signs of A-232 intoxication, which occurred between 1:07 and 9:08 (h:min) after exposure (See results). At this time, the AHAK solution tested (AHAK_220_, AHAK_0_ or AHAK_880_) was applied. In the AHAK_880_ efficacy evaluation experiment this occurred in all animals before the end of the predetermined maximal 3-h exposure time (see results). Accordingly, no SSPD was performed in this experiment before returning the animals to their home cage for follow-up after treatment commenced. Conversely, as described above, in the AHAK_220_ treatment efficacy evaluation experiment, SSPD was performed in all animals at three hours post exposure, before moving the animals to follow up cages. This was done because the experimenters were blind to the type of AHAK solution (AHAK_220_ or AHAK_0_) used in the different animals (see “Study design” below).

## Study design

### Characterization of the intoxication following dermal exposure to a lethal dose of A-232

To determine a lethal A-232 dermal dose to be used in the AHAK treatment efficacy evaluation experiments, as well as characterize the intoxication signs and dynamics of their appearance following this dose, 6 pigs (weight (mean ± SEM) 10.95 ± 0.26 kg) were used. n = 6 was chosen to allow identification of differences in survival of ≥ 66% (4/6 animals) between treated and non-treated animals exposed to a lethal dose of A-232 in treatment evaluation experiments (see below) as significant (*p* < 0.05), using a Fisher’s Exact-test. Preliminary toxicity studies in mice showed that the i.v. LD_50_ dose of A-232 was about half that of VX (unpublished data). Accordingly, all 6 pigs were dermally exposed to a dose of 2 mg/kg A-232, half the VX dermal lethal dose (4 mg/kg) determined in our previous study (Bloch-Shilderman et al. 2023). All animals exposed to this dose for 3 h followed by SSPD died, leading to determination of 2 mg/kg as a lethal dermal dose of A-232 for a 3-h exposure, to be used in the AHAK treatment efficacy evaluation experiments.

### AHAK_220_/AHAK_0_/AHAK_880_ efficacy evaluation experiments

The first AHAK formulation tested for efficacy was AHAK_220_. For this purpose, 12 pigs were randomly allocated into one of two experimental groups: 1. Dermal exposure to a lethal dose of A-232 (2 mg/kg, see above), followed by i.m. TAB upon onset of obvious signs of intoxication coupled with dermal application of AHAK_220_ at the site of exposure (AHAK_220_, n = 6, weight (mean ± SEM) 10.57 ± 0.65 kg). 2. Identical to group 1, except for application of a mock AHAK solution instead of AHAK_220_ (AHAK_0_, n = 6, weight (mean ± SEM) 10.66 ± 0.58 kg). This group served as a Sham AHAK_220_ application control group. Each experimental session consisted of two animals, one from each group, with the experimenters blind as to the type of AHAK formulation (AHAK_220_ or AHAK_0_) used in each of the animals. In both groups, pending recurrence of intoxication signs after TAB administration, animals were given repeated i.m. administrations of TAB (for more details see “Antidotes administration”).

In a second experiment, the efficacy of the AHAK_880_ formulation was tested in two groups of animals: 1. Dermal exposure to a lethal dose of A-232 (2 mg/kg), followed by i.m. TAB upon onset of obvious signs of intoxication coupled with dermal application of AHAK_880_ at the site of exposure (TAB-AHAK_880_, n = 6, weight 9.77 ± 0.25 kg). 2. Identical to group 1, except for administration of TA instead of TAB (TA-AHAK_880_, n = 6, weight 10.05 ± 0.24 kg). In both groups, pending recurrence of intoxication signs after TAB/TA administration, animals were treated with repeated administration of atropine and midazolam (for more details see “Antidotes administration”).

In both experiments, exposure duration was up to 3 h. Following SSPD (AHAK_220_ efficacy evaluation experiment) or AHAK_880_ application (AHAK_880_ efficacy evaluation experiment), the animals were moved to designated cages for follow-up. All animals were further treated with antidotes according to their clinical condition (monitored continuously for at least 14 h post initiation of exposure) as described above. Typical signs of A-232 intoxication monitored and recorded were loss of appetite, disorientation, tremor, fasciculation, salivation, respiratory distress, unconsciousness, and death. Treatment was ceased if symptoms did not reappear for at least 4 h following the last treatment. Additional clinical evaluation was performed at 24 h and every 12 h thereafter until full behavioral recovery of the animal, defined as absence of observable intoxication signs coupled with normal home cage behavior and appetite. Whole-blood samples for determination of ChE activity relative to baseline (pre-exposure) were obtained at 0.5, 1, 2, 3, 5, 7, 9, 24, 48 and 72-h post exposure, and then once a week for 6 weeks. In the experiment comparing the TAB-AHAK_880_ and TA-AHAK_880_ groups, an additional blood sample was taken immediately before treatment. Following full recovery and throughout the 6 weeks of follow-up after the experiment, no clinical signs, abnormal home cage behavior or changes in appetite were observed in any of the animals, inspected twice daily by the staff of the center for preclinical studies at the IIBR, and by the experimenters at the times of blood withdrawal for determination of ChE activity listed above.

In both experiments, n = 6 per experimental group was chosen to allow identification of differences in survival of ≥ 66% (4/6 animals) between treatment groups and in comparison to non-treated animals as significant (*p* < 0.05), using a Fisher’s Exact-test.

### Evaluation of late AHAK_880_ application efficacy

This experiment aimed at evaluating whether AHAK_880_ would be beneficial against dermal exposure to Novichok CWAs, even if applied with a significant delay relative to appearance of intoxication signs (an elaboration regarding the rational for this experiment is provided in the relevant “Results” section). For that purpose, pigs (n = 6) were randomly assigned into two experimental groups: 1. Dermal exposure to a low dose (in the range of 0.33–0.41 mg/kg) of A-232, followed by Late dermal Application (LA) of AHAK_880_ at the site of exposure 12 h post A-232 application (hereafter LA-AHAK_880_, n = 3, weight 11.00 ± 0.65 kg). 2. Identical to group 1, but without AHAK_880_ application (hereafter No Application (NA), n = 3, weight 8.83 ± 0.43 kg). Two animals, one from each experimental group, served in each experimental session. Exposure duration was 3 h, followed by SSPD as described above. n = 3 animals per group was chosen based on a power analysis, to allow identification of an effect size of 2 and larger as significant (*p* < 0.05) using a mixed model ANOVA with two groups and 23 repeated measurements, with a power of 0.8 (see “Statistical analyses” below).

Whole-blood samples for determination of ChE activity relative to baseline (pre-exposure) were obtained at 0.5, 1, 2, 3, 5, 7, 9, 12, 24, 36, 48, 60, 72, 84, 96, 108 and 120 h post exposure, and as of 1 week post exposure once a week for 6 weeks. Following the exposure and throughout the 6 weeks of follow-up after the experiment, no clinical signs, abnormal home cage behavior or changes in appetite were observed in any of the animals, inspected twice daily by the staff of the center for preclinical studies at the IIBR, and by the experimenters at the times of blood withdrawal for determination of ChE activity as described above.

Summary of the different experimental groups used in the study and their experimental timeline is provided in Table 1.

**Table 1:** Summary of experimental groups and timeline

Group name	n	A-232 dose (mg/kg)	Initial treatment^	SSPD*	Repeated treatment^ upon recurrence of intoxication signs	Blood ChE activity assessment times post exposure
1. Lethal dose intoxication Characterization	**6**	**2**	**–**	√	**–**	0 (pre-exposure), 0.5, 1, 2, 3, 5, 7, 9 h ^#^
2. AHAK_0_	**6**	**2**	TAB + AHAK_0_ upon obvious intoxication signs	√	TAB	0 (pre-exposure), 0.5, 1, 2, 3, 5, 7, 9, 24 h ^#^
3. AHAK_220_	**6**	**2**	TAB + AHAK_220_ upon obvious intoxication signs	√	TAB	0 (pre-exposure), 0.5, 1, 2, 3, 5, 7, 9, 24, 48 and 72 h 1, 2, 3, 4, 5, 6 weeks
4. TA-AHAK_880_	**6**	**2**	TA + AHAK_880_ upon obvious intoxication signs	**–**	Atropine (0.07 mg/kg) Midazolam (0.22 mg/kg) upon convulsions or loss of consciousness	0 (pre-exposure), 0.5, 1, 2, 3, 5, 7, 9, 24, 48 and 72 h. An additional sample was taken immediately before first treatment 1, 2, 3, 4, 5, 6 weeks
5. TAB-AHAK_880_	**6**	**2**	TAB + AHAK_880_ upon obvious intoxication signs	**–**	Atropine (0.07 mg/kg) Midazolam (0.22 mg/kg) upon convulsions or loss of consciousness	Same as TA-AHAK_880_ group
6. NA	**3**	0.33–0.41	**–**	√	**–**	0 (pre-exposure), 0.5, 1, 2, 3, 5, 7, 9, 12, 24, 36, 48, 60, 72, 84, 96, 108 and 120 h 1,2,3,4,5,6 weeks
7. LA- AHAK_880_	**3**	0.33–0.41	AHAK_880_ 12 h after exposure initiation	√	**–**	Same as NA group

^ All antidotes were administered i.m. TAB: TMB-4, 1.14 mg/kg; atropine, 0.07 mg/kg; benactyzine, 0.09 mg/kg. TA: TMB-4, 1.14 mg/kg; atropine, 0.07 mg/kg (for more details see "Antidotes administration"). All AHAK solutions were applied dermally at the site of A-232 exposure (for more details see ‘AHAK_220_/AHAK_0_/AHAK_880_ dermal application’). *Skin Surface Physical Decontamination, conducted 3h after exposure initiation (for more details see "Percutaneous A-232 exposure procedure"). √ denotes applied. – denotes not applied. ^#^ In the Lethal dose characterization and AHAK_0_ groups, all animals died by 24 and 26h post exposure initiation, respectively. Accordingly, the latest blood sampling for ChE activity assessment conducted in these groups was at 9 and 24h, respectively (see results).

### Statistical analyses

Statistical analyses were conducted using the SPSS software (Version 29). Graphs were created using the Prism 9.5 software (GraphPad Software, Inc.). Survival ratios following different treatments were compared using a Fisher’s exact test. Comparisons of time to first treatment, whole-blood ChE activity level relative to baseline at this time, number of recurrences of intoxication signs following the exposure, total number of antidotes administered, treatment duration and time to full recovery between treatment groups were conducted using a student’s t test. Comparison of whole-blood ChE activity in the LA-AHAK_880_ and NA groups was conducted using a mixed model ANOVA, with time post-exposure as a repeated within-subject factor and the two groups as a between-subject factor, followed by an analysis of simple main effects. All statistical analyses were conducted blindly regarding the identity of the different treatment groups. Results were considered significant for *p* < 0.05.

## Results

### Clinical signs of intoxication and their timeline of appearance, following percutaneous exposure to a lethal dose (2 mg/kg) of A-232

The time to onset of observable intoxication signs in 6 animals exposed dermally to 2 mg/kg of A-232 is summarized in Table 2.

**Table 2:** Intoxication signs and their onset time, following percutaneous exposure to 2 mg/kg of A-232

Intoxication signs	Individual time* to onset post initiation of exposure
Mastication	0:30^3^, 1:26^6^, 1:32^5^, 2:01^2^, 2:18^4^, 3:03^1^
Salivation	0:30^3^, 0:35^4^, 1:27^6^, 1:57^5^, 2:25^2^, 7:07^1^
Tremor	1:15^5^, 1:27^6^, 2:00^3^, 2:18^4^, 3:18^2 #^
Convulsions	1:35^6^, 1:50^5^, 2:12^3^, 3:18^4^, 5:20^2^, 8:21^1^
Respiratory distress	1:55^6^, 2:13^5^, 2:35^3^, 4:24^4^, 5:53^2^, 14:20^1^
Death	2:12^6^; 2:23^5^, 2:56^3^, 5:56^4^, 10:53^2^, 20:35^1^

**Fig. 1:**
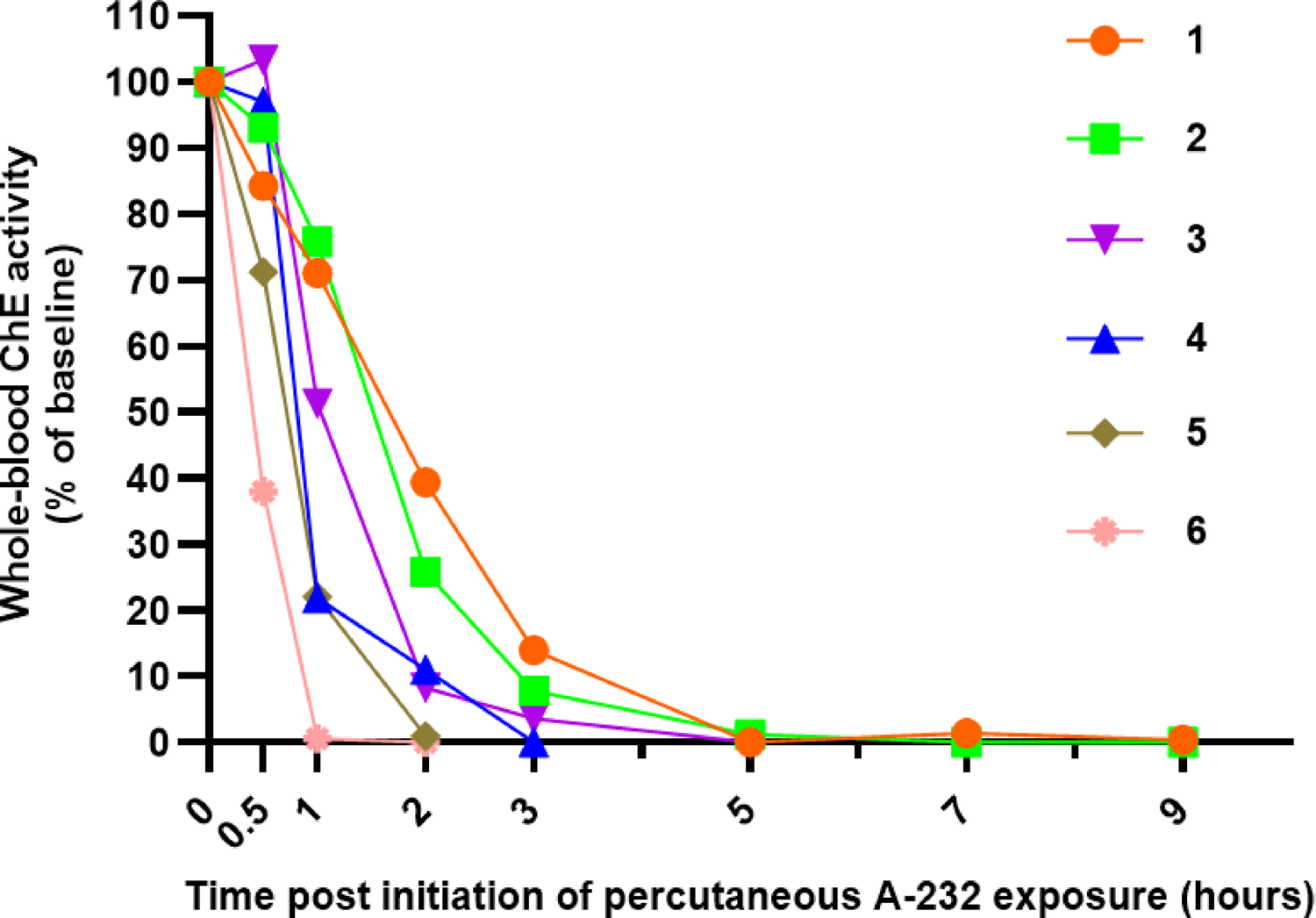
Whole-blood ChE activity following percutaneous exposure to a lethal dose of A-232. Pigs (n = 6/group) were dermally exposed to A-232 (2 mg/kg) for 3 h. Whole-blood ChE activity (% relative to baseline) of all animals at different time points following beginning of exposure is presented

Awake (unanesthetized) pigs (n = 6) were percutaneously exposed to an A-232 dose of 2 mg/kg for 3 h. All animals` times to onset of observable intoxication signs post initiation of exposure are displayed. Each animal has been assigned an identifying number from 1 to 6, that appears in uppercase to the right of the data referring to it in all table rows. The same identifying number is also used in Fig. 1. e.g. animal 3 displayed mastication and salivation at 0:30, tremor at 2:00, convulsions at 2:12, etc. The whole-blood ChE activity data for this animal is shown in purple (animal 3) in Fig. 1. *All times are given as hrs:min. ^#^ Animal 1 did not show tremor.

In the first 30 min after A-232 dermal application, pigs did not exhibit observable signs of intoxication. Mastication and salivation were detected as early as 0:30 post application, albeit with a wide range of appearance, up to 3:03 and 7:07, respectively (unless otherwise specified, all times are given as hrs:min). Tremor appeared at 1:15 post-application, with a 1:15–3:18 range of appearance. Generally, the more severe intoxication signs appeared later, and were also characterized by a wide range of appearance. Specifically, convulsions and respiratory distress were observed between 1:35 to 8:21 and 1:55 to 14:20 post A-232 application, respectively. Death occurred between 2:12 to 20:35 post application.

The blood ChE activity profile of the above 6 animals is depicted in Fig. 1. As evident from the figure, the decrease in whole-blood ChE activity following the exposure (for all animals except animal 6) was characterized by two phases: a rapid phase during the first one to three hours from initiation of exposure, followed by a slower phase thereafter. The whole-blood ChE activity of all animals was ≤ 1.0% relative to baseline as of 1–5 h after initiation of exposure. The differences between animals in the rate of whole-blood ChE activity reduction were generally in line with the rate of appearance and escalation of intoxication signs.

Taken together, the above results demonstrate that percutaneous A-232 intoxication is characterized by a large variability in the rate of appearance and escalation of intoxication signs. Additionally, the continued reduction in whole-blood ChE activity observed in three of the 6 animals (see animals 1–3 in Fig. 1) after SSPD (conducted 3 h after A-232 application, see above), indicates continuous penetration of A-232 into the blood from a (most likely dermal) depot of the agent formed by the exposure. These characteristics are reminiscent of those observed following dermal exposure to VX (e.g., Chilcot et al. 2003), highlighting that the benefit of eliminating the dermal depot using AHAK in the case of percutaneous VX intoxication (Bloch-Shilderman et al. 2023), may highly likely also apply to percutaneous A-232 intoxication.

### The efficacy of repeated TAB antidotal treatments combined with AHAK_220_ application, against percutaneous poisoning by a lethal dose of A-232

**Fig. 2:**
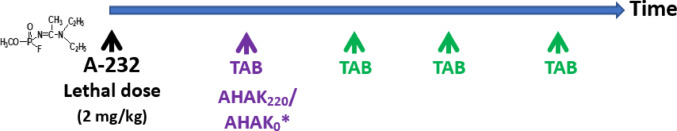
Percutaneous A-232 intoxication and treatment timeline. Pigs (n = 12, 6 per group) were exposed to a lethal dose (2 mg/kg) of A-232 between the shoulder blades for up to 3 h. At the onset of clear observable clinical signs of intoxication (either 25 min of continuous tremor and intensifying mastication coupled with mild salivation, or severe salivation, or convulsions, or respiratory distress, the first to occur) animals were treated i.m. with TAB (TMB-4, 1.14 mg/kg; Atropine, 0.07 mg/kg; Benactyzine, 0.09 mg/kg,) and immediately afterwards dermally applied with either AHAK_220_ or *AHAK_0_ (a solution similar in color and texture to the AHAK_220_ solution, albeit with no active ingredient capable of hydrolyzing A-232) at the site of exposure. The experimenters were blind to the type of AHAK solution (AHAK_220_/AHAK_0_) used in the different animals. All animals were decontaminated with SSPD at the end of the three-hour exposure and treated with repeated i.m. TAB injections in the case of recurrence of intoxication signs. For more details regarding the different treatments and experimental procedure see “Materials and methods”

In order to study the potential benefit of AHAK_220_ dermal application as an adjunct to conventional antidotal treatment against percutaneous A-232 intoxication, we examined the contribution of dermal application of the AHAK_220_ solution to repeated TAB administration following percutaneous poisoning by the above-described lethal dose (2 mg/kg) of A-232. The experimental timeline is detailed in Fig. 2.

The time to first treatment relative to the beginning of exposure and two parameters indicative of the clinical status of the animals in the two experimental groups (AHAK_220_/AHAK_0_) at this time, the clinical indication for treatment and percent of whole-blood ChE activity level relative to baseline, are listed in Table 3. The clinical signs of intoxication which served as an indication for treatment were identical in the AHAK_220_ and AHAK_0_ experimental groups. Additionally, the time to appearance of these signs and accordingly the time to first treatment, as well as the residual whole-blood ChE activity at this time, did not significantly differ between the two groups (t (10) = 0.753, *p* = 0.469 and t (10) = 0.505, *p* = 0.624 for time to first treatment and % of whole-blood ChE activity relative to baseline, respectively). Hence, the data presented in Table 3, coupled with the fact that the experimenters were blind to the type of AHAK solution used in the different animals, indicates that any differences in outcome between the two groups may be attributed to differences in efficacy between the two AHAK solutions.

**Table 3:** Time post A-232 dermal exposure initiation, intoxication signs and whole-blood ChE activity at first treatment, in the AHAK_220_ and AHAK_0_ groups

Group	Mean time to first treatment (Individual data)	Clinical indication to treat (fraction of animals)	Mean whole-blood ChE activity at first treatment* (Individual data)
AHAK_220_	2:04 (0:56^5^, 1:20^1^, 1:20^3^, 1:34^4^, 2:28^2^, 4:49^6^)	25 min of continuous mild signs (2/6)^1,2^ Convulsions (4/6)^3–6^	< 4.83 (0^5^, 2^6^, < 3^4^, < 7^3^, < 8^2^, < 9^1^)
AHAK_0_	2:55 (1:07^3^, 1:21^2^, 1:27^1^, 1.54^5^; 5.04^4^, 6:37^6^)	25 min of continuous mild signs (2/6)^2, 3^ Convulsions (4/6)^1, 4–6^	< 6.33 (0^5^, < 1^4^, < 2^6^, < 8^1^, < 12^2^, 15^3^)

Pigs (n = 6/group) were dermally exposed to A-232 and treated as described in Fig. 1. The mean and individual data for time (hrs:min) to first treatment relative to the beginning of exposure, and for *percent of whole-blood ChE activity relative to baseline at this time (in both groups, for animals whose time to first treatment occurred between designated blood sampling times for whole-blood ChE activity assessment, whole-blood ChE activity at first treatment was documented as < value measured for the last sample collected before treatment), as well as the clinical indication for treatment, in the AHAK_220_ and AHAK_0_ groups, are presented. In both groups (i.e. in each row), each animal has been assigned an identifying number from 1 to 6 that appears in uppercase to the right of the data referring to it in all table columns. e.g., the time to first treatment in animal 5 in the AHAK_220_ group was 0:56, due to an indication to treat of convulsions. No residual whole-blood ChE activity (0) was measured for this animal at this time.

**Fig. 3:**
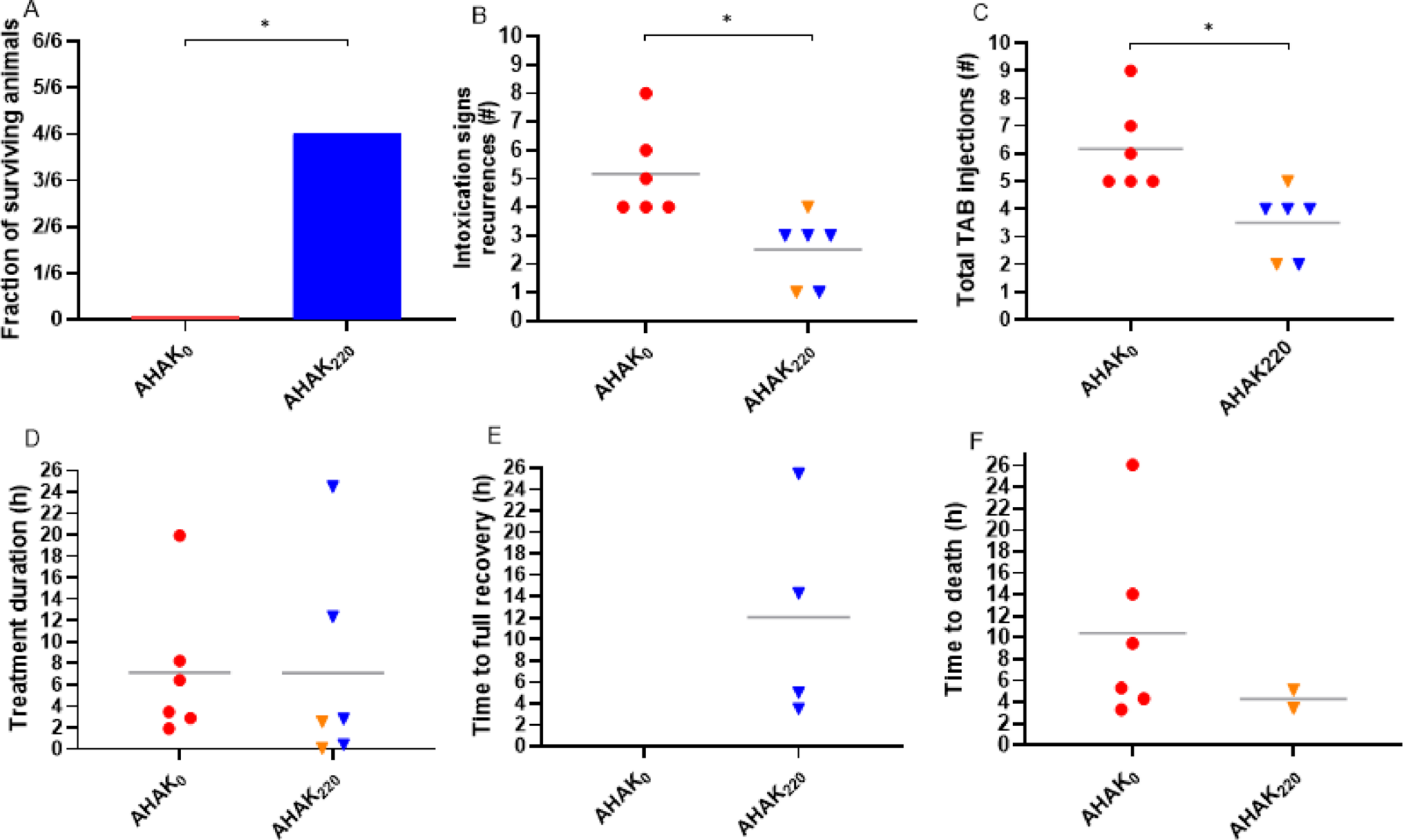
Parameters of interest indicative of the therapeutic efficacy observed in the AHAK_0_ and AHAK_220_ experimental groups. Pigs (n = 6/group) were dermally exposed to A-232 (2 mg/kg) and treated as described in Fig. 2. **A** Fraction of surviving animals, **B** The number of recurrences of intoxication signs, **C** Total TAB injections administered, **D** Treatment duration, **E** Time to full recovery and **F** Time to death, for animals in the AHAK_0_ and AHAK_220_ groups. In figures B-F, AHAK_0_ animals are represented by red circles, AHAK_220_ animals that survived by blue triangles and AHAK_220_ animals that died by orange triangles. The line parallel to the x axis for each of the groups represents the group mean. Note that mild signs of intoxication such as lack of appetite and mild tremor, which were observed for varying periods following the last TAB treatment given in AHAK_220_ animals that survived, were not considered as an indication for treatment, hence the difference between treatment duration and time to full recovery (defined as complete absence of intoxication signs, coupled with normal home-cage behavior and appetite) in these animals. *p < 0.05

Different parameters of interest indicative of the therapeutic efficacy observed in the two experimental groups are depicted in Fig. 3. The most prominent finding presented in Fig. 3 is that despite an average of 6.16 administrations of TAB, none (0/6) of the animals dermally applied with AHAK_0_ survived, while 4/6 of the animals dermally applied with AHAK_220,_ which received on average only 3.5 administrations of TAB, survived (Fig. 3a, c. *p* = 0.03 for difference in survival ratio (Fisher’s Exact Test). t (10) = 3.24, *p* = 0.009 for number of TAB administrations). This correlated with a smaller number of intoxication signs recurrence events in the AHAK_220_ as compared to the AHAK_0_ animals (Fig. 3b. t (10) = 3.24, *p* = 0.009). These findings are in line with degradation of an A-232 dermal depot by AHAK_220_, and accordingly a reduction of A-232 penetration into the bloodstream, which in 4/6 of the animals applied with AHAK_220_ enabled survival.

**Fig. 4:**
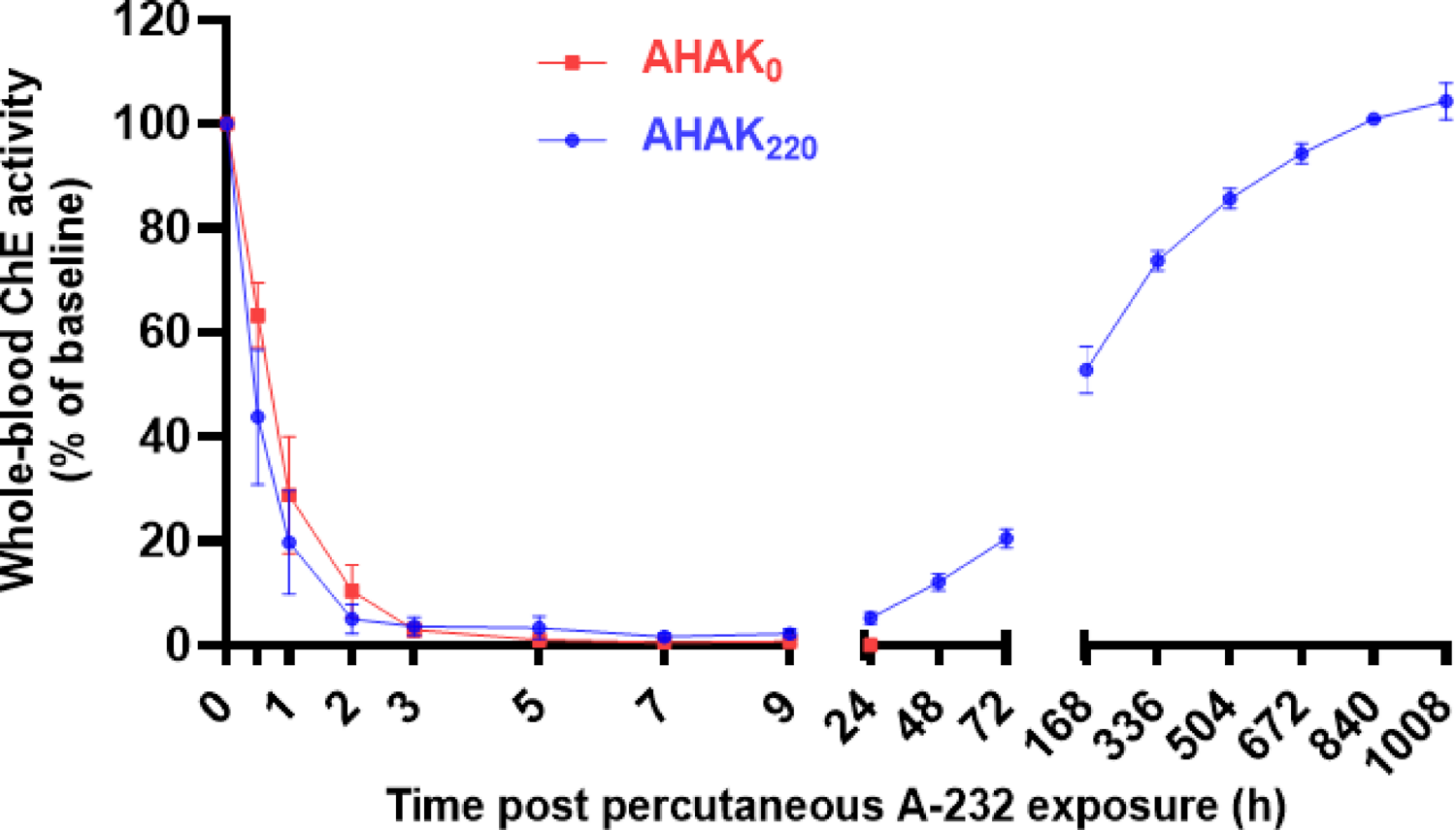
Whole-blood ChE activity following percutaneous A-232 intoxication in the AHAK_0_ and AHAK_220_ experimental groups. Pigs (n = 6/group) were dermally exposed to A-232 and treated as described in Fig. 2. Whole-blood ChE activity (Mean ± SEM of % relative to baseline) in the AHAK_0_ and AHAK_220_ experimental groups at different time points following beginning of exposure is presented

The whole-blood ChE activity profile in the two experimental groups is given in Fig. 4. As evident from the figure, whereas in animals applied with AHAK_0_ a continuous reduction in average whole-blood ChE activity was observed until death of all animals by ~ 26 h post exposure, in the 4 animals applied with AHAK_220_ that survived, recovery in whole-blood ChE activity was observed beginning 24 h post exposure, with a return to baseline level (Mean ± SEM, 100.99 ± 0.23) at 5 weeks (840 h) post exposure. Note that the rise in whole-blood ChE above baseline levels observed at 6 weeks post exposure has been observed previously following exposure to other OPNAs in various species (Bloch-Shilderman et al. 2018, 2023).

### The efficacy of standard antidotal treatments combined with AHAK_880_ application, against percutaneous poisoning by a lethal dose of A-232

**Fig. 5:**
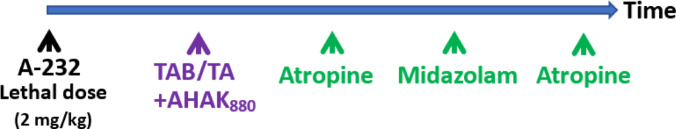
Percutaneous A-232 intoxication and treatment timeline in the AHAK_880_ efficacy evaluation experiment. Pigs (n = 12, 6/group) were exposed to a lethal dose (2 mg/kg) of A-232 between the shoulder blades for up to 3 h. At the onset of clear observable clinical signs of intoxication (for details see Fig. 2 legend), animals were treated with TAB (TAB-AHAK_880_ group) or with TA (TA-AHAK_880_ group). Immediately afterwards, all animals were dermally applied with the AHAK_880_ lotion at the site of exposure. Upon recurrence of intoxication signs, all animals were further treated with atropine and midazolam according to their clinical condition (for more details see "Materials and methods")

As described above, addition of AHAK_220_ dermal application to conventional antidotal treatment following percutaneous intoxication by a lethal dose of A-232 provided a significant contribution to survival, but did not enable survival of all exposed animals. Accordingly, we next examined whether a saturated AHAK solution (hereafter AHAK_880_) containing the maximal concentration of the potassium salt of AHA soluble in the DMSO/H_2_O 1:4 solvent used (880 mg/ml, for more details see “Materials and methods”), may further improve survival. The experimental timeline is detailed in Fig. 5.

The time to first treatment relative to the beginning of exposure, the clinical indication for treatment, and whole-blood ChE activity level (percent relative to baseline) at this time, in the TA-AHAK_880_ and TAB-AHAK_880_ experimental groups, are listed in Table 4. The indication to treat was generally similar in both groups - 25 min of continuous mild signs in most (4/6) and all (6/6) animals in the TA-AHAK_880_ and TAB-AHAK_880_ groups, respectively. The time to appearance of these signs and accordingly the time to first treatment, as well as the residual whole-blood ChE activity at this time, did not significantly differ between the two groups (t (10) = 0.44, *p* = 0.67 and t (10) = 0.28, *p* = 0.79 for time to first treatment and % of whole-blood ChE activity relative to baseline, respectively).

**Table 4:** Time post dermal A-232 exposure initiation, intoxication signs and whole-blood ChE activity at first treatment, in the TA-AHAK_880_ and TAB-AHAK_880_ groups

Group	Mean time to first treatment (Individual data)	Clinical indication to treat (Fraction of animals)	Mean whole-blood ChE activity at first treatment* (Individual data)
TA-AHAK_880_	2:00 (1:29^3^; 1:34^6^, 2:05^1^, 2:14^2^, 2:14^5^, 2:27^4^)	25 min of continuous mild signs (4/6)^1, 3, 5, 6^ Respiratory distress (2/6)^2, 4^	11.3^#^ (2^3^, 3^5^, 4^4^, 9^1^, 12^2^, 38^6^)
TAB-AHAK_880_	2:05 (1:43^2^, 2:05^3^, 2:08^4^, 2:08^6^, 2:13^1^, 2:14^5^)	25 min of continuous mild signs (6/6)^All animals^	9.5^#^ (4^1^, 4^3^, 6^6^, 8^2^, 8^5^, 27^4^)

Pigs (n = 6/group) were dermally exposed to A-232 and treated as described in Fig. 5. The mean and individual data for time (hrs:min) to first treatment relative to the beginning of exposure, and for percent of whole-blood ChE activity *relative to baseline at this time, as well as the clinical indication for treatment in the TA-AHAK_880_ and TAB-AHAK_880_ experimental groups, are presented. ^#^The whole blood ChE activity relative to baseline of one animal in each of the TA-AHAK_880_ and TAB-AHAK_880_ groups at the time of first treatment was markedly higher than that of all other animals in its respective group (38 and 27% for the odd animal in the two groups, respectively). The mean (range) whole-blood ChE activity values at this time for the majority of animals (5/6, excluding the data of these two animals) in the TA-AHAK_880_ and TAB-AHAK_880_ groups were 6 (range 2–12) and 6 (range 4–8), in the two groups, respectively. In both groups (i.e. in each row), each animal has been assigned an identifying number from 1 to 6, that appears in uppercase to the right of the data referring to it in all table columns. e.g., The time to first treatment in animal 3 in the TA-AHAK_880_ group was 1:29, due to an indication to treat of 25 min of continuous mild signs. Whole-blood ChE activity of this animal at this time was 2% of baseline.

**Fig. 6:**
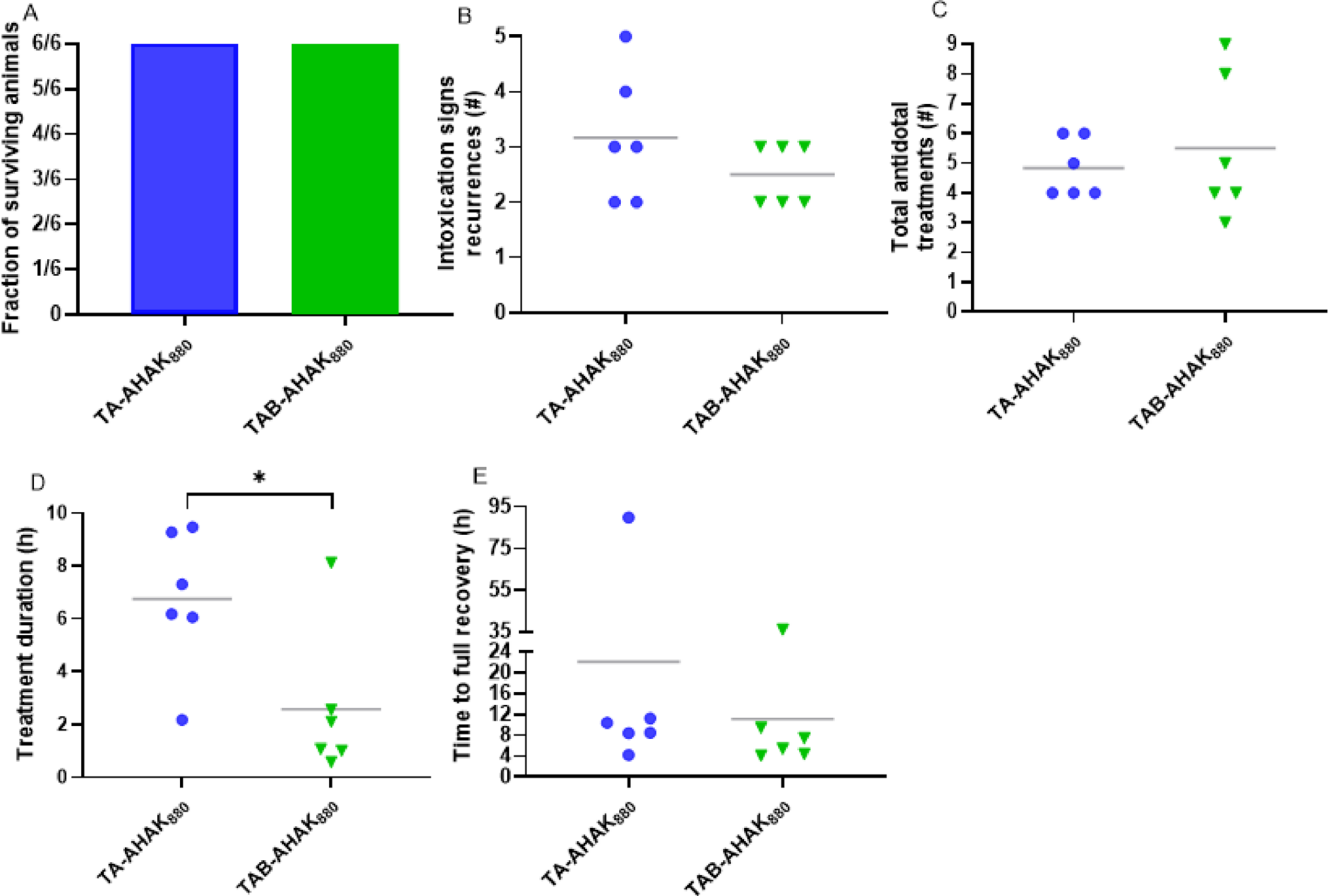
Parameters of interest indicative of the therapeutic efficacy observed in the TA-AHAK_880_ and TAB-AHAK_880_ groups. Pigs (n = 6/group) were dermally exposed to a A-232 and treated as described in Fig. 5. **A** Fraction of surviving animals, **B** The number of recurrences of intoxication signs , **C** Number of antidotal treatments administered, **D** Treatment duration and **E** Time to full recovery, for animals in the TA-AHAK_880_ and TAB-AHAK_880_ groups. In figures B-E, TA-AHAK_880_ animals are represented by blue circles and TAB-AHAK_880_ animals by green triangles. The line parallel to the x axis for each of the groups represents the group mean. Note that mild signs of intoxication such as lack of appetite and mild tremor, which were observed for varying periods following the last antidotal treatment given, were not considered as an indication for treatment, hence the difference between treatment duration and time to full recovery (defined as complete absence of intoxication signs, coupled with normal home-cage behavior and appetite). *p < 0.05

Different parameters of interest indicative of the therapeutic efficacy observed in the two groups are depicted in Fig. 6. Both treatment regimens led to survival of all animals (Fig. 6a), demonstrating the high efficacy of combining AHAK_880_ with standard antidotal treatment against percutaneous intoxication by a lethal dose of A-232. Notably, although the number of recurrences of intoxication signs in both groups did not significantly differ (mean ± SEM = 3.17 ± 0.48 and 2.5 ± 0.22 in the TA-AHAK_880_ and TAB-AHAK_880_ groups, respectively, t(10) = 1.26,  *p* = 0.235, Fig. 6b), treatment duration was significantly shorter in the TAB-AHAK_880_ as compared to the TA-AHAK_880_ group (mean ± SEM = 2.42 ± 1.08 and 6:54 ± 1.07 in these two groups, respectively, t (10) = 2.63, *p* = 0.025, Fig. 6d).

**Fig. 7:**
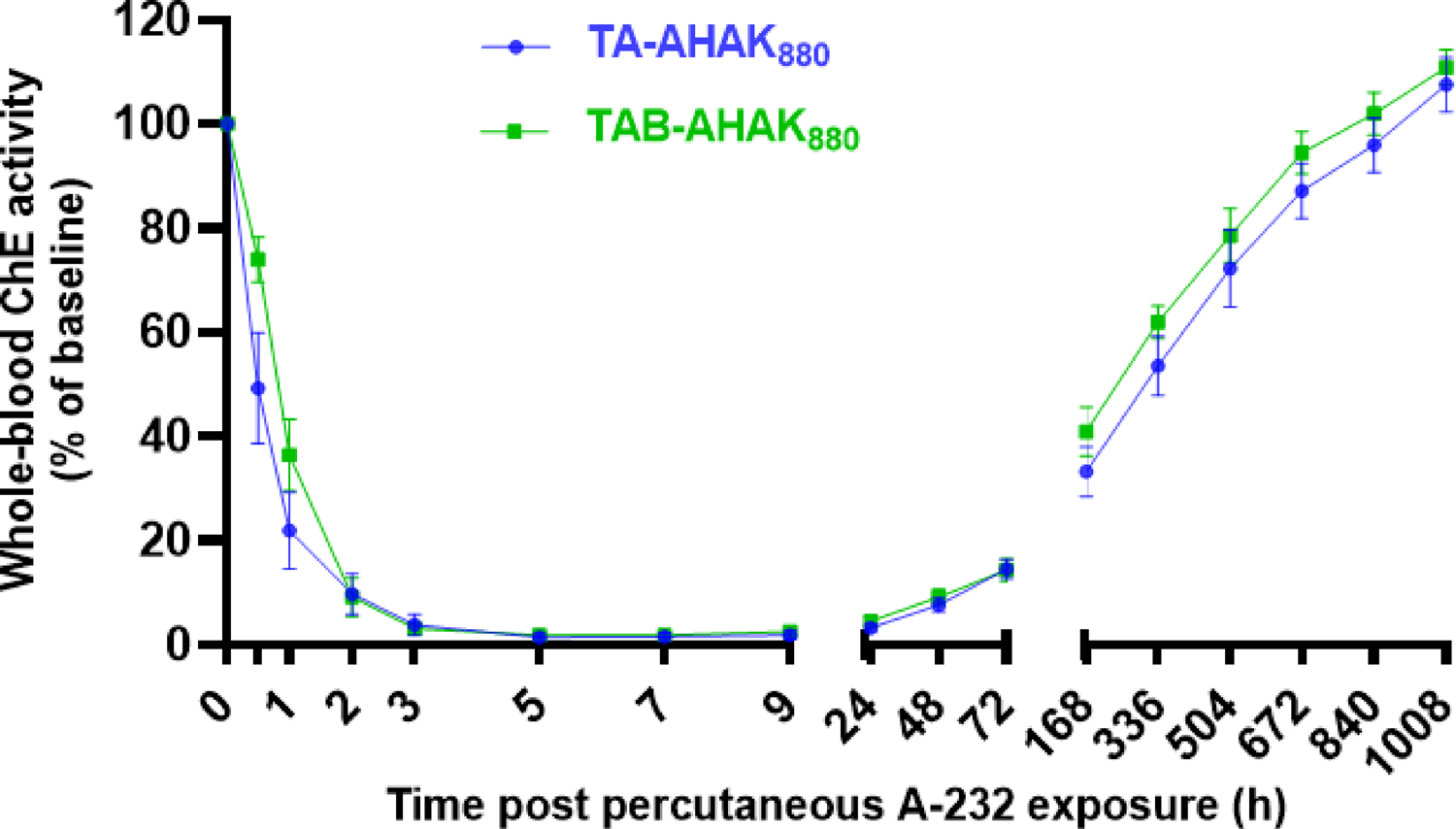
Whole-blood ChE activity following percutaneous A-232 intoxication in the TA-AHAK_880_ and TAB-AHAK_880_ groups. Pigs (n = 6/group) were dermally exposed to A-232 and treated as described in Fig. 5. Whole-blood ChE activity (Mean ± SEM of % relative to baseline) in the TA-AHAK_880_ and TAB-AHAK_880_ groups at different time points following beginning of exposure is presented

The whole-blood ChE activity profile in the two groups is given in Fig. 7. Similarly to AHAK_220_ animals that survived (see Fig. 4), a clear recovery in whole-blood ChE activity was observed in both groups as of 24h post exposure, with a return to baseline level at 5 and 6 weeks (840 and 1008h) post exposure in the TAB-AHAK_880_ and TA-AHAK_880_ groups, respectively (Mean ± SEM 101.94 ± 4.13 and 107.6 ± 5.2, respectively).

**Fig. 8:**
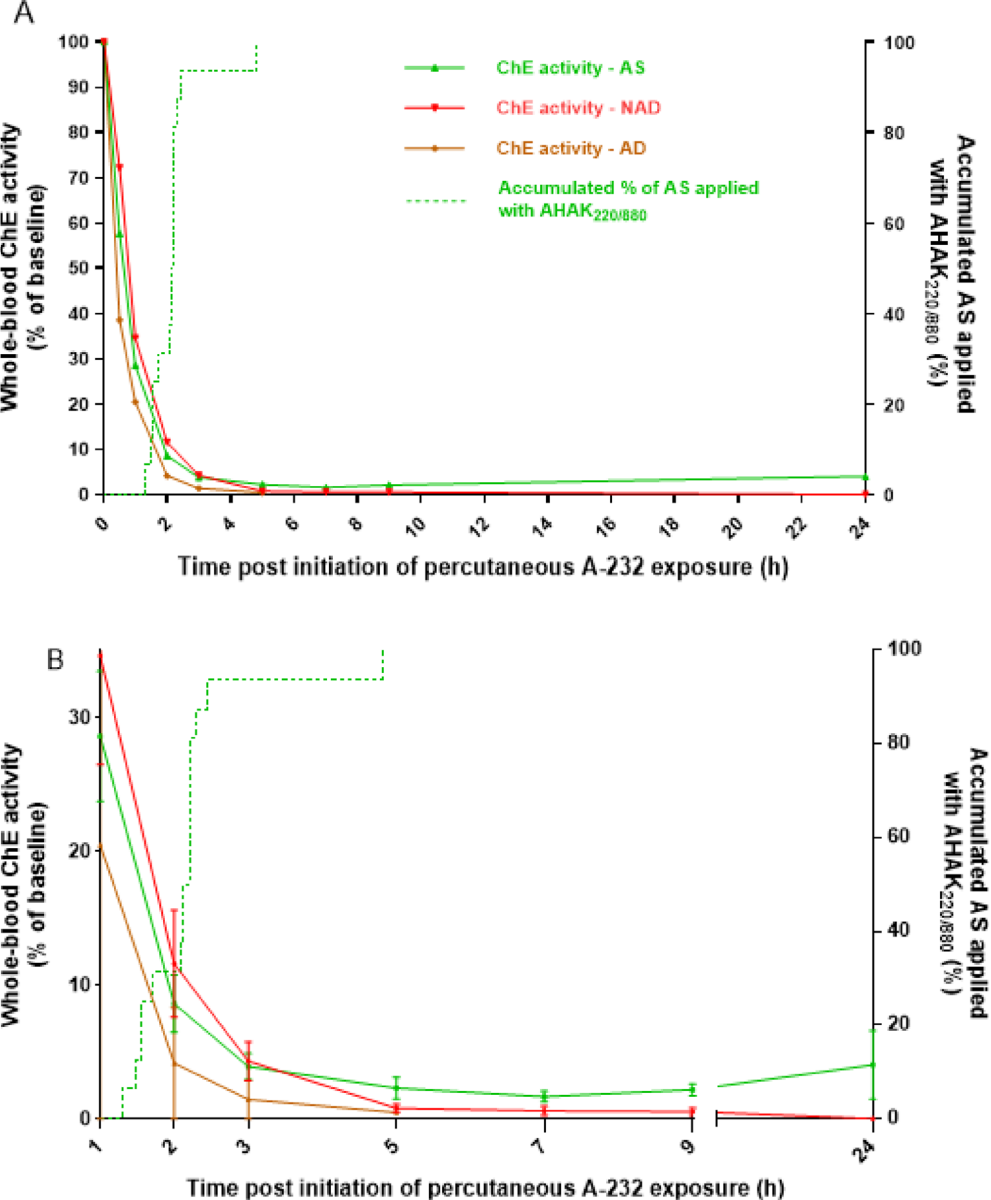
Whole-blood ChE activity in AS, NAD and AD animals, as well as cumulative percent of AS animals dermally applied with AHAK_220_/AHAK_880_, following percutaneous A-232 intoxication. Pigs were dermally exposed to a lethal dose (2 mg/kg) of A-232 and treated as described above. **a** Whole-blood ChE activity (Mean ± SEM of percent relative to baseline) of AS (n = 16, green line), NAD (n = 12), red line) and AD (n = 2, brown line) animals (refers to the left Y axis), as well as cumulative percent of AS animals already applied with AHAK_220_/AHAK_880_ (dashed green line, refers to the right Y axis), during the 24h following beginning of exposure. **b** Magnification of the 1-9h and the 24h data of whole-blood ChE activity and cumulative percent of AS animals already applied with AHAK_220_/AHAK_880_ presented in A

As described above, the whole-blood ChE activity of all non-treated animals exposed to a lethal dose (2 mg/kg) of A-232 was ≤ 1.0% relative to baseline as of 1–5 h after initiation of exposure (see Fig. 1), and the differences between animals in the rate of whole-blood ChE activity reduction were generally in line with the rate of appearance and escalation of intoxication signs. A similar pattern of reduction in whole-blood ChE activity was observed in AHAK_0_ animals. Accordingly, in the case that AHAK_220_/AHAK_880_ dermal application enabled survival by eliminating a dermal depot of A-232 formed following the exposure (thus decreasing the amount of A-232 that entered the circulation), this might be reflected in a different pattern of whole-blood ChE activity reduction in AHAK_220_/AHAK_880_ animals that survived, as compared to non-treated animals and animals applied with AHAK_0_ that died. Concurrently, such a difference, if exists, should not be observed in the two AHAK_220_ animals that died. In order to explore this possibility, we examined the relation between the pattern of whole-blood ChE activity reduction following the exposure in these different groups, and the time to AHAK_220_/AHAK_880_ application in surviving animals (Fig. 8). The above groups consisted of: **1**. 4/6 AHAK_220_, 6/6 TA-AHAK_880_ and 6/6 TAB-AHAK_880_ animals (i.e. 4 surviving animals of group 3 and all animals of groups 4 and 5 listed in Table 1), hereafter AHAK_220_/AHAK_880_ Applied that Survived (AS) group, n = 16. **2**. 6/6 non-treated animals from the lethal dose intoxication characterization experiment and 6/6 AHAK_0_ animals (i.e. all animals of groups 1 and 2 listed in Table 1), hereafter Non AHAK_880_/AHAK_220_ Applied that Died (NAD) group, n = 12. **3**. 2/6 AHAK_220_ animals (i.e. 2 non-surviving animals of group 3 listed in Table 1), hereafter AHAK_220_ Applied that Died (AD) group, n = 2.

As evident from Fig. 8a, at low resolution, the pattern of reduction in whole-blood ChE activity following the exposure in the AS group (continuous green line) till 9 h post exposure, does not look markedly different than that in the NAD group (continuous red line). However, closer examination of the first 1–9 h following exposure reveals a difference between these two groups (Fig. 8b). Specifically, in the first 2 h following exposure, the whole-blood ChE activity dropped with a similar slope in the two groups, with the residual activity somewhat lower in the AS as compared to the NAD group. However, between 2 and 3 h post exposure, the slope of whole-blood ChE activity reduction became more moderate in the AS as compared to the NAD group, such that at 3 h post exposure the residual whole-blood ChE activity in the two groups was similar. This trend continued, and accordingly as of 5 h post exposure, at all time-points sampled the residual whole-blood ChE activity in the AS group was higher than that in the NAD group. Furthermore, as of 9 h post exposure whole-blood ChE activity in the AS group began to rise, while in the 5/12 (41.66%) surviving NAD animals at that time it continued to decline, until death of all NAD animals by 24h post exposure. Notably, the above-described moderation in the slope of whole-blood ChE activity reduction in the AS animals, which became evident between 2 and 3 h following exposure, coincided with the time by which 15/16 (93.75%) of these animals had been dermally applied with AHAK_220_/AHAK_880_ (see Fig. 8b). Taken together, the above data are in line with degradation of a dermal depot of A-232 by AHAK_220_/AHAK_880_, which in 16/18 animals dermally applied with these lotions decreased the amount of A-232 that entered the circulation so as to enable survival. The failure of AHAK_220_ application to afford survival in one of the two AD animals was likely due to very rapid penetration of A-232 into the circulation (no residual whole-blood ChE activity was observed in this animal at the time of first treatment/AHAK_220_ application 56 min from beginning of exposure), such that by the time AHAK_220_ was applied it was too late to allow for sufficient degradation of A-232 so as to prevent death. The reason for the inability of AHAK_220_ to enable survival in the second AD animal, which at the time AHAK_220_ was applied (2:28 from beginning of exposure) had residual whole-blood ChE activity of 8% relative to baseline, is unclear.

### The effect of late AHAK_880_ dermal application on whole-blood ChE activity, following percutaneous exposure to a low dose of A-232

We next examined whether AHAK_880_ would still be beneficial against dermal exposure to Novichok CWAs, even if dermally applied with a significant delay relative to the appearance of intoxication signs. As demonstrated above, following dermal exposure to a lethal dose of A-232, conventional antidotal treatment alone, initiated upon appearance of intoxication signs, was not sufficient to enable prolonged survival (see AHAK_0_ treated animals). Hence, in a real-life situation of such exposure, in which time to identification of the cause of intoxication and implementation of ideal designated treatment practices may be long delayed, intensive medical intervention measures such as mechanical ventilation would likely need to be implemented in order to enable survival (e.g. Steindl et al. 2021). Nevertheless, the above data indicate that even in such a scenario, decomposition of a skin depot of the Novichok agent is expected to significantly shorten the duration of continued penetration of the agent from the skin into to the circulation, and accordingly the time during which such intensive medical intervention measures would need to be implemented. Since applying intensive medical intervention measures such as prolonged mechanical ventilation was beyond the scope of our experimental setup, we could not test the efficacy of late AHAK_880_ application using a lethal dose of A-232. Hence, as an alternative, we examined the effect of late (12 h post intoxication) AHAK_880_ application on the whole-blood ChE activity recovery profile following dermal exposure to a low dose of A-232, as a proxy for the expected effects of late AHAK_880_ application on the recovery rate following exposure to a lethal dose of the agent.

**Fig. 9:**
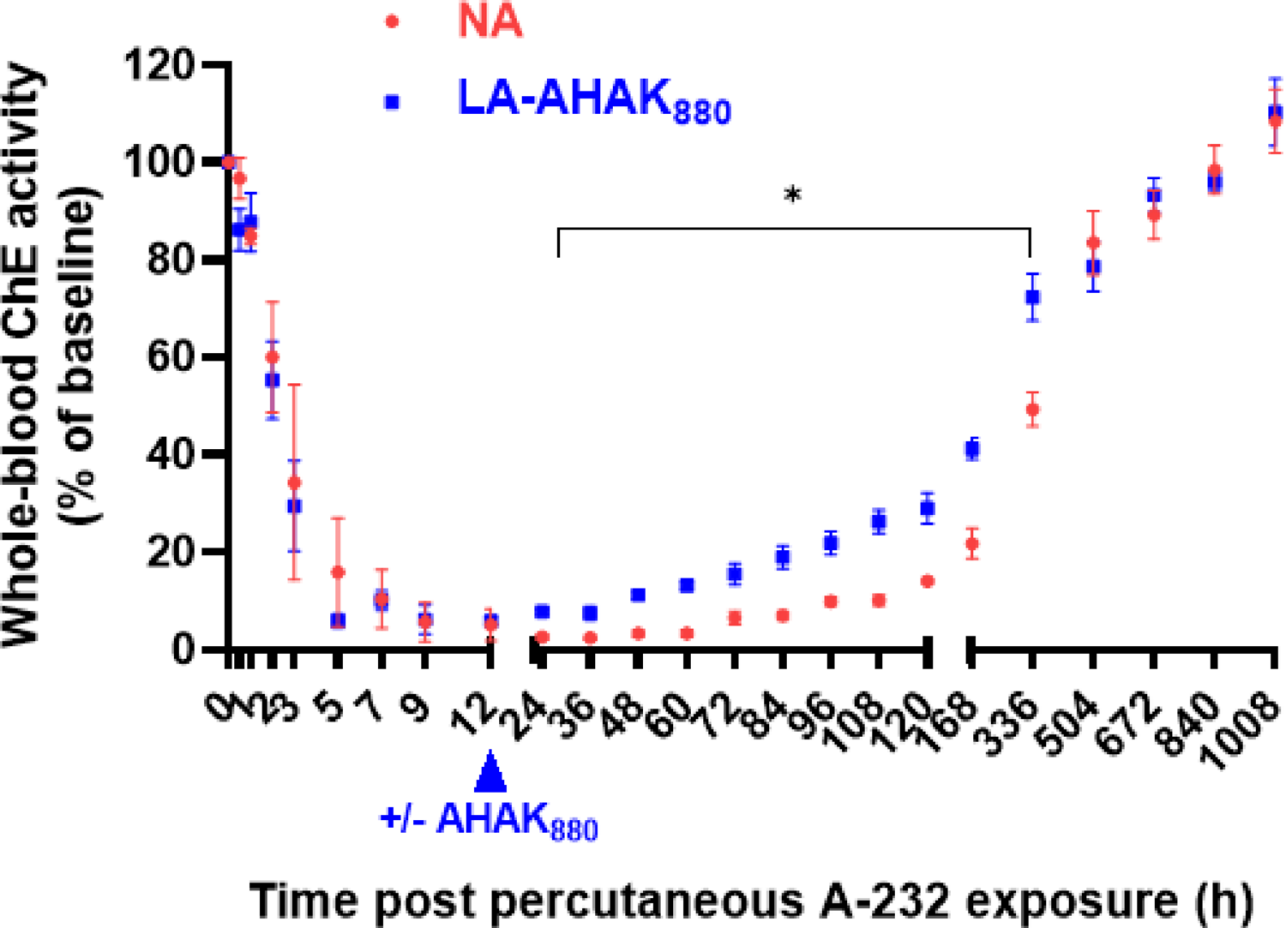
Whole-blood ChE activity following percutaneous exposure to a low dose of A-232 and late AHAK_880_ treatment. Pigs (n = 3/group) were dermally exposed to a low dose of A-232 (0.33–0.41 mg/kg) for three hours, followed by SSPD (for more details see  "Materials and methods"). 12 h post exposure initiation, AHAK_880_ was dermally applied at the site of exposure in one group (Late Application (LA) of AHAK_880_, hereafter LA-AHAK_880_ grup blue rectangles). No Application (NA) of AHAK_880_ was conducted in the second group (hereafter NA group, red circles). Whole-blood ChE activity (mean ± SEM of percent relative to baseline) in both groups at different time points following the beginning of exposure is presented. *p < 0.05

The experimental timeline is detailed in Fig. 9. An analysis of whole-blood ChE activity in the two experimental groups at different time points following the exposure revealed a significant group by time interaction (F (23) = 1.652, *p* = 0.49). Specifically,  until 12 h following beginning of exposure, the time of AHAK_880_ dermal application in the LA-AHAK_880_ group, the residual whole-blood ChE activity did not significantly differ between the LA- AHAK_880_ and NA groups. However, at 24 h and at all succeeding time points until 2 weeks (336 h) post exposure, activity in the LA-AHAK_880_ group was significantly higher than that in the NA group. These findings are also consistent with degradation of a dermal A-232 depot by AHAK_880_, leading to a faster recovery of whole-blood ChE activity in the LA-AHAK_880_ group. They therefore suggest that even delayed AHAK_880_ treatment may accelerate recovery following percutaneous poisoning with A-232.

## Discussion

Here we characterize, to the best of our knowledge for the first time, the intoxication following percutaneous exposure to a lethal dose of the Novichok CWA A-232, using the swine model considered as the most adequate animal model for human percutaneous absorption research (Simon and Maibach 2000; Chilcott et al. 2003; Dorandeu et al. 2007; Fémy et al. 2023). In addition to a thorough description of the acute clinical course following the exposure, we also demonstrate the inability of conventional antidotal treatment against OP CWAs by itself, to protect against such an exposure. Conversely, we show that combining such treatment with dermal application of a low (AHAK_220_) or high (AHAK_880_) dose of AHAK (the potassium salt of the FDA approved medication AHA in DMSO/H_2_O 1:4) at the site of exposure, leads to survival of most and all animals, respectively. Finally, we demonstrate that late AHAK_880_ application also exerts a beneficial effect, by speeding up the time to ChE activity recovery.

The onset of symptoms following percutaneous exposure to a lethal dose of A-232 was slow (though highly variable between animals: tens of minutes to several hours), and their evolution gradual, with a typical sequence of appearance: Mastication (range of appearance (hrs:min) 0:30–3:03), salivation (0:30–7:07) and tremor (1:15–3:18) were the first to appear, followed by convulsions (1:35–8:21). All animals then developed respiratory distress (1:55–14:20), characterized by a pronounced contraction of abdominal muscles synchronized with respiration, generally accompanied by copious secretions, which exacerbated over time to bouts of gasping and ultimately led to death (2:12–20:35). Reduction in whole-blood ChE activity following the exposure was characterized by a rapid phase during the first one to three hours after initiation of exposure, followed by a slower phase thereafter. A ≥ 99% reduction in whole-blood ChE activity was observed in all animals 1–5 h after initiation of exposure, with the differences between animals in the rate of whole-blood ChE activity reduction generally in line with the rate of appearance and escalation of intoxication signs. Due to the above-described large variability in the time to onset of intoxication signs following the exposure, the trigger to treat in the current study was based on clinical condition (obvious observable signs of intoxication, for more details see "Materials and methods") rather than time following exposure, as will most likely also be the case in a real-life scenario of percutaneous exposure to Novichok CWAs (or any other OP CWA). In this regard, the escalating respiratory distress observed, which in a real-life situation will likely necessitate respiratory aid and ultimately mechanical ventilation to allow survival, is in line with the prolonged mechanical ventilation of exposed individuals reported in the Salisbury and Navalny incidents (OPCW; Steindl et al. 2021).

The slow onset and gradual development of intoxication signs, as well as the large variability between animals in these parameters observed in this study, are reminiscent of findings from previous studies of percutaneous VX exposure in rats (Bloch-Shilderman et al. 2019), guinea pigs (Joosen et al. 2010), swine (Chilcott et al. 2003; Hamilton et al. 2004; Bloch-Shilderman et al. 2023) and men (Munro et al. 1994; Sim 1962). Based on these and additional studies that documented accumulation of VX inside the skin (Craig et al. 1977; Wetherell et al. 2008; Thors et al. 2107, 2021), prolonged intoxication, and a need for recurrent antidotal treatment following percutaneous exposure to VX (e.g. van der Schans et al. 2003; Chilcott et al. 2005a, 2005b; Joosen et al. 2010, 2013, 2017; Rolland et al. 2013; Rice et al. 2015; Bloch-Shilderman et al. 2019, 2023), it is widely accepted that after absorption into the skin, VX forms a dermal reservoir (depot) from which it slowly penetrate into the bloodstream. Accordingly, a therapeutic strategy termed ‘catch-up therapy’, by which reactive molecules are delivered into the skin in order to neutralize the VX dermal depot before it reaches the bloodstream, has been suggested (Chilcott et al. 2005a). In our previous in-vial and ex-vivo studies, we demonstrated that AHAK may both efficiently degrade VX and quickly penetrate the skin (Nahum et al. 2021). We further showed that when combined with standard antidotal treatment against OP CWAs following percutaneous exposure to a lethal dose of VX in swine, it leads to a significant reduction in intoxication signs recurrence and shortening of the medical supervision duration needed, most likely by acting as such a ‘catch-up therapy’ (Bloch-Shilderman et al. 2023). Additionally, we demonstrated the ability of AHAK and its different derivatives to efficiently decompose Novichok CWAs such as A-232, A-234 and A-230, in reaction conditions and a time frame suitable for acting as a ‘catch-up therapy’ also against these agents (Smolkin et al. 2024).

As described above, intoxication signs onset times following percutaneous exposure to a lethal dose of A-232 observed in the present study were generally longer and had a wider range than those observed following a similar exposure to a lethal dose of VX (Bloch-Shilderman et al. 2023), indicative of slower penetration of A-232 as compared to VX from the skin into the blood circulation. Additionally, whereas VX inhibited AChE is readily reactivated by oximes (Munro et al 1994; Worek et al 1998; Aurbek et al. 2006), Novichok CWA inhibited AChE exhibits high resistance to oxime mediated reactivation (Steindl et al. 2021; Hrabinova et al. 2024; Kovarik et al. 2025). Furthermore, Novichoks are highly stable against hydrolysis at physiological pH (Harvey et al. 2020; deKoning et al. 2022; Smolkin et al. 2024). Hence, taken together, the above highlight an even higher potential benefit of eliminating a skin depot formed following percutaneous exposure to A-232, than that observed following a similar exposure to VX (Bloch-Shilderman et al. 2023). Indeed, following percutaneous exposure to a lethal dose of VX, repeated antidotal treatment alone was sufficient to save all exposed animals, albeit with significantly more intoxication signs recurrence events and a significantly longer medical supervision duration as compared to the same treatment combined with AHAK (*Ibid*). Conversely, in the present study, following percutaneous exposure to a lethal dose of A-232, addition of AHAK_220_/AHAK_880_ to antidotal treatment was essential to enable survival. Specifically, while none of the animals percutaneously exposed to a lethal dose of A-232 and treated with repeated conventional antidotes against OP CWAs and mock AHAK (AHAK_0_ group) survived, 4 of 6 animals treated with repeated antidotes and the less concentrated lotion AHAK_220_ (AHAK_220_ group), and all 12 animals treated with repeated antidotes and the saturated AHAK lotion (TAB-AHAK_880_ and TA-AHAK_880_ groups), survived. This correlated with less recurrences of intoxication signs in the surviving AHAK_220_ as well as all TA-AHAK_880_ and TAB-AHAK_880_ treated animals, as compared to the AHAK_0_ treated animals (mean ± SEM = 2.00 ± 0.81, 3.17 ± 0.48 and 2.5 ± 0.22 vs. 5.16 ± 2.11 in these 4 groups, respectively, Figs. 3b and 6b).

In line with the documented resistance of Novichok CWAs inhibited AChE to reactivation by oximes (Steindl et al. 2021; Hrabinova et al. 2024; Kovarik et al. 2025), no rise in whole-blood ChE activity was observed at any stage after the exposure in the AHAK_0_ group (Fig. 4), despite an average of > 6 administrations of the oxime TMB-4 as part of the repeated TAB treatment used in these animals. Conversely, in the surviving AHAK_220_/AHAK_880_ dermally applied animals (AS group), a deceleration in the rate of whole-blood ChE activity reduction relative to non-treated and AHAK_0_ treated animals (NAD group) was apparent as of 2h and intensified until 5h post exposure, in congruence with the time of AHAK_220_/AHAK_880_ treatment in these animals (Fig. 8). Furthermore, a rise in whole-blood ChE activity was observed in this group as of 9 h post exposure. These results are in line with degradation of a dermal depot of A-232 by AHAK_220_/AHAK_880_, leading to a reduction in the amount of A-232 that entered the circulation and consequently in whole-blood ChE inhibition. In this regard, it is noteworthy that although whole-blood ChE activity is not a direct measure of OP CWA toxicity, which is induced by inhibition of tethered synaptic AChE in target organs, the reduction in whole-blood ChE activity following OP CWA intoxication is generally well correlated with toxicity symptoms (e.g., Chilcott et al. 2003; Bloch-Shilderman et al. 2018, 2023 in the case of VX). This is because as long as blood ChE is available to bind the nerve agent, it may serve as a scavenger reducing its binding to synaptic AChE at target organs (Rosenberg and Saxena 2020). Hence, although the absolute differences in residual whole-blood ChE activity between the AS and NAD groups in the 5–9 h time window from beginning of exposure were small (Fig. 8), these small differences likely correlated with sufficient vs. insufficient residual AChE activity in target organs to allow survival, in the AS and NAD groups, respectively. Incidentally, as in the swine the vast majority of whole blood ChE is AChE (96%, Naik et al. 2012), the whole blood ChE activity measured in this study mostly represents AChE activity.

In regard to the above, it is noteworthy that due to recurrences of intoxication signs, repeated treatments were still necessary to afford survival in AS animals, likely due to a time lag between AHAK_220_/AHAK_880_ application and sufficient degradation of the A-232 dermal depot. Accordingly, it is possible that addition of a persistent bioscavenger capable of neutralizing A-232 (such as exogenous ChE, e.g. Noy-Porat et al. 2015) to the immediate antidotal treatment given upon appearance of intoxication signs, may neutralize A-232 that will enter the circulation in the time window between AHAK application and degradation of the dermal depot, thus negating the need for repeated treatments. Nevertheless, due to a limited dose of such bioscavenger that may be used, which might not be sufficient to neutralize all the Novichok agent residing in the skin depot, this will not negate the need for elimination of the dermal depot by AHAK.

Last, the faster recovery of whole-blood ChE activity observed following late (12 h post beginning of exposure) AHAK_880_ treatment after exposure to a low dose of A-232, as compared to the absence of AHAK treatment, is also in line with degradation of a dermal depot of A-232 by AHAK (Fig. 9).

To sum, our results demonstrate that percutaneous exposure to the Novichok A-232 is characterized by slow appearance and exacerbation of intoxication signs, and that these reoccur after a period of mitigation following conventional antidotal treatment against OP CWAs. As is believed to be the case with VX, this likely stems from a faster absorption of A-232 into the skin than from the skin into the blood stream, leading to formation of a dermal depot of A-232 from which the agent slowly penetrates into the bloodstream, causing a slowly developing and sustained intoxication. We further show that as with VX (Bloch-Shilderman et al. 2023), the use of AHAK as a ‘catch-up’ therapy intended to enable both skin surface A-232 decontamination and decomposition of the A-232 dermal depot, coupled with standard antidotal treatment, has a significant beneficial effect. Moreover, whereas in the case of VX this beneficial effect is expressed as a reduction in the number of repeated antidote administrations and shortening of the time to recovery, and accordingly the medical supervision duration needed (*Ibid*), in the case of A-232 the addition of AHAK to conventional antidotal treatment, administered upon appearance of intoxication signs, is obligatory and sufficient to afford survival in the absence of respiratory support. Stated differently, this indicates that if given concurrently with conventional antidotal treatment upon intoxication signs onset, AHAK may negate the need for respiratory support and a lengthy hospitalization period to allow survival (e.g. Steindl et al 2021). Additionally, we demonstrate that AHAK is expected to exert a beneficial effect also following late application, by degrading and thus speeding up the time to depletion of the A-232 dermal depot, and accordingly the time to ChE activity recovery.

Hence, taken together, our results are the first demonstration of the beneficial effect of a ‘catch-up therapy’ against percutaneous intoxication by an OP CWA of the Novichok family. Furthermore, together with our previous results regarding the contribution of AHAK to countering percutaneous intoxication by VX (Bloch-Shilderman et al. 2023), they delineate the aptness of AHAK to serve as a generic medical countermeasure against percutaneous intoxication by persistent low-volatility OP CWAs of both the V and Novichok families. This is of importance as in a real-life scenario of percutaneous intoxication by an OP CWA, an unequivocal identification of the OP CWA used will likely be long delayed (e.g. Steindl et al. 2021). Based on the above results and on the demonstration of the safety of prolonged (24 h) whole body application of the AHAK lotion (Bloch-Shilderman et al. 2023), AHAK has been approved for emergency use against dermal exposure to persistent low-volatility OP CWAs in Israel.

## Abbreviations

AChE: Acetylcholinesterase
AHA: Acetohydroxamic acid
AHAK: The potassium salt of AHA in DMSO/H_2_O 1:4
ChE: Cholinesterase
CWA: Chemical warfare agent
OP: Organophosphorus
TAB: TMB4, Atropine and benactyzine
TA: TMB4, Atropine
SSPD: Skin surface physical decontamination
